# Optimization of tunable guided-mode resonance filter based on refractive index modulation of graphene

**DOI:** 10.1038/s41598-019-56194-4

**Published:** 2019-12-27

**Authors:** Hwa-Seub Lee, Joon Young Kwak, Tae-Yeon Seong, Gyu Weon Hwang, Won Mok Kim, Inho Kim, Kyeong-Seok Lee

**Affiliations:** 1 Center for Electronic Materials, Korea Institute of Science and Technology, Seoul, 02792 Korea; 2 Department of Materials Science and Engineering, Korea University, Seoul, 02841 Korea; 3 Department of Nanomaterials Science and Engineering, Korea University of Science and Technology, Daejeon, 34113 Korea

**Keywords:** Materials for optics, Theory and computation, Optical materials and structures, Materials science, Nanoscience and technology, Optics and photonics

## Abstract

To fabricate a tunable optical filter with a fast response in the near infrared region, a tunable guided-mode resonance (GMR) filter using graphene was proposed and its performance was optimized. In this study, a rigorous coupled wave analysis method was employed to systematically investigate the effects of geometrical configuration of graphene-integrated GMR filters and the optical properties of constituent materials including graphene on their spectral response in terms of tunability and extinction ratio. It was found that as the graphene is located close to the waveguide and the evanescent-field strength at the interface increases, the GMR filter exhibits better tunability. The bandwidth of the filter could be drastically reduced by adopting a low-index contrast grating layer, so that the extinction ratio of an optical signal could be greatly improved from 0.91 dB to 27.99 dB as the index contrast decreased from 0.99 to 0.47, respectively. Furthermore, new practical device designs, that is easy to fabricate and effectively implement the electric-field doping of graphene at low gate voltage, were also suggested and theoretically validated. These results demonstrate not only the excellent potential of a graphene-based tunable GMR filter but also provide practical design guidelines for optimizing the device performance.

## Introduction

A guided-mode resonance (GMR) filter is an optical component that filters light through an optical interaction between a zero-order grating with a period smaller than the incident wavelength and an adjacent waveguide^1,2^. In a GMR filter, only the 0^th^-order diffraction component of a specific wavelength that satisfies a phase-matching condition can produce a resonance band in transmission or reflection spectra. Higher-order components are coupled into the waveguide modes. The guided modes are coupled out into the radiation mode interacting with the grating again, which results in a high efficiency at resonance. The GMR filter has excellent out-of-band rejection and has a very narrow bandwidth with a high efficiency of almost 100%. In addition, the resonance wavelength highly depends on the geometrical parameters, such as the period of the diffraction grating, the duty cycle, the optical thickness of the waveguide layer, and the refractive index of a surrounding material^3,4^. Due to these characteristics, GMR filters have attracted much attention in many practical applications such as narrow band-stop filters^5,6^, optical polarizer^7^, and biosensors^8,9^.

Various attempts have been also made to give active tunability to GMR filters. Most of them replace bulk components of GMR filter with photo-isomerizable polymers^10,11^, thermo-optic^12^ or electro-optic materials^13,14^. However, since the refractive index of these materials are not significantly modulated, operation based on the bulk response is necessary to obtain an adequate tuning range. This limits the response time or prevents cost-effective fabrication of the device. Fang *et al*.^15^, suggested a low-cost gradient-period-grating-based tunable GMR filter whose spectral resonance wavelength is tuned mechanically by changing the illumination position on the device. This approach is still far from solving the fundamental issues.

Therefore, there is a need to apply a material which exhibits a large change in the refractive index and the fast response to external stimuli without relying on the bulk effect. Graphene could be a promising candidate. Graphene is a two-dimensional, planar material consisting of a single layer of carbon atoms, which has excellent electrical conductivity and low optical loss. In addition, its optical constants can be altered drastically when exposed to external stimuli such as light, electric-field, heat, and chemical doping^16,17^. Therefore, its integration into the GMR filter could produce an active device with high sensitivity and fast switching of resonance wavelength. Recently, a graphene-based active tunable GMR filter has been proposed^18^.

The dielectric constant of graphene can be derived from the Kubo formula representing the surface conductivity given by the following Eq. (1)^19^:

1 $$\sigma ({\mu }_{c})=\frac{j{e}^{2}(\omega -j{\tau }^{-1})}{\pi {\hslash }^{2}}\times [\begin{array}{c}\frac{1}{{(\omega -j{\tau }^{-1})}^{2}}{\int }_{0}^{{\rm{\infty }}}E(\frac{{\rm{\partial }}{f}_{d}(E,{\mu }_{c},T)}{{\rm{\partial }}E}-\frac{{\rm{\partial }}{f}_{d}(\,-\,E,{\mu }_{c},T)}{{\rm{\partial }}E})dE\\ \,-\,{\int }_{0}^{{\rm{\infty }}}\frac{{f}_{d}(\,-\,E,{\mu }_{c},T)-{f}_{d}(E,{\mu }_{c},T)}{{(\omega -j{\tau }^{-1})}^{2}-4{(E/\hslash )}^{2}}dE\end{array}].$$

The first term in Eq. (1) denotes the intraband response, and the second term is the interband response. *E* is the energy, τ is the scattering time of electron related to quality of graphene (1/2τ = Γ is the electron relaxation energy), *T* is the temperature, *μ*_*c*_ is the chemical potential of graphene (or Fermi level shift), *ω* is the angular frequency, *ħ* is the reduced Planck’s constant, *e* is the electron charge, and ${f}_{d}(E,{\mu }_{c},T)=({e}^{(E-{\mu }_{c})}/{k}_{B}T)+1{)}^{-1}$ is the Fermi-Dirac distribution (*k*_B_ is Boltzmann’s constant). It should be noted that if the values of *T* and τ^−1^ are fixed in Eq. (1), the surface conductivity of graphene σ_G_ can be modified depending on *μ*_c_, which is easily controlled by external stimuli. The in-plane permittivity of graphene is related with the surface conductivity using following equation^20^:

2 $${\varepsilon }_{G}={\varepsilon }_{x}={\varepsilon }_{y}=1+\frac{{\sigma }_{G}}{\omega {\varepsilon }_{0}{t}_{G}}.$$

In Eq. (2), *ε*_0_ is the vacuum dielectric constant and *t*_G_ (=0.34 nm) is the thickness of the monolayer graphene.

**Figure 1:**
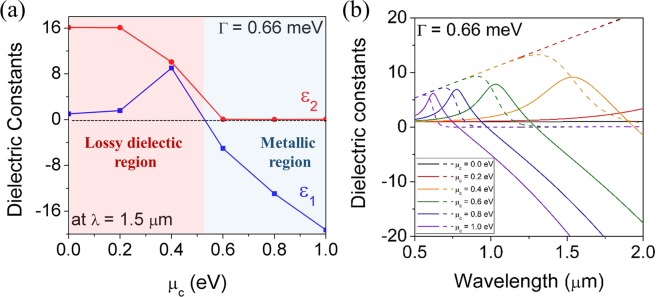
(**a**) The behavior of the complex permittivity of graphene as a function of μ_c_ at the wavelength of 1.5 μm and (**b**) the behavior of the complex permittivity of graphene as a function of wavelength for different μ_c_ values. Here, *T* = 300 K, and Γ = 0.66 meV, respectively. Solid line and dotted line indicate *ε*_1_ and *ε*_2_, respectively.

Figure 1 exhibits the complex dielectric constants of graphene calculated from Eqs. (1) and (2) as functions of chemical potential and wavelength, assuming that *T* = 300 K and *Γ* = 0.66 meV. Figure 1(a) shows the dependence of the real part *ε*_1_ and the imaginary part *ε*_2_ of the dielectric constant on the chemical potential of graphene for the wavelength *λ* = 1.5 μm. The *ε*_1_ has positive values at *μ*_c_ smaller than 0.5 eV and peaks at 0.4 eV. Then it crosses zero and turns to a negative value when *μ*_c_ exceeds 0.5 eV, exhibiting a dielectric-to-metal transition. Graphene reveals an absorptive characteristic in the dielectric region, as evidenced by the large *ε*_2_ related with optical loss. On the other hand, the *ε*_2_ becomes negligible in the metallic region indicating low-loss nature of graphene. Figure 1(b) displays the spectral dispersion relations of the complex permittivity of graphene as a function of the chemical potential. It is shown that the transition from lossy-dielectric to quasi-metal with low-loss depends predominantly on the *μ*_c_ value and is gradually blue-shifted with increasing *μ*_c_. When *μ*_c_ is larger than 0.6 eV, graphene behaves as a low-loss metal in the near-infrared communication band of 1.3 to 1.55 μm. Since the dielectric constant of graphene undergoes a tremendous change depending on the *μ*_c_ value, it is expected that a tunable optical filter with a fast response and large tunability can be realized by incorporating graphene as an active component of GMR filter.

In this study, we simulated the effects of geometrical parameters of GMR filter and optical properties of constituent materials including graphene layer on the spectral response of the tunable filter using a rigorous coupled wave analysis (RCWA) method. We systematically and theoretically compared the device performance of different configurations in terms of the tunability and extinction ratio of signal channel. On the basis of the results obtained, the key factors that determine the performance of the tunable GMR filter and the design rules for optimizing the performance were suggested.

## Results and Discussion

### Geometrical configurations of simulation model

The GMR filter can be classified into two types, namely, a single layer GMR and a multilayer GMR depending on whether a grating and a waveguide layer are separately provided^2^. In a single layer GMR, the grating layer also serves as a waveguide layer. Because of the open structure of the waveguide core region, the single layer GMR structure is sensitive to a change in the bulk environment, but the degree of freedom of designing properties is limited. On the other hand, for the multilayer GMR, the waveguide and grating layers are separately provided. The multilayer GMR structure has high degree of freedom of designing, which is advantageous in optimizing the filter spectrum. For example, the filter can have very low sideband reflectance by designing the grating to be an antireflection layer. The filter linewidth can also be very easily adjusted by controlling the index contrast of grating.

**Figure 2:**
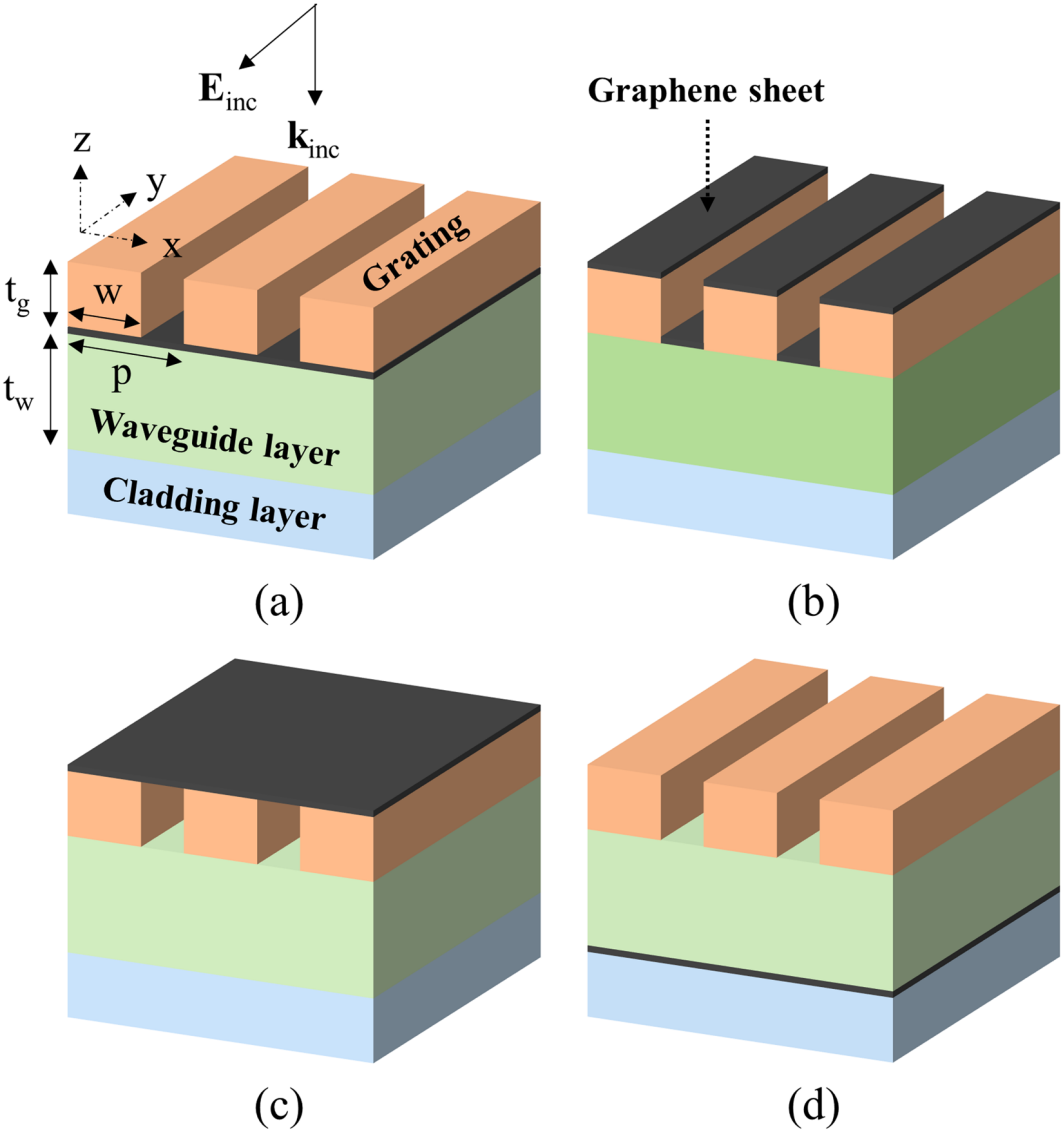
Configurations of four different multilayer guided-mode resonance filters illuminated by TE-polarized light; The graphene (**a**) located between the grating and waveguide layers, (**b**) covers the grooves and ridges of the diffraction grating, (**c**) supported on the top surfaces of the grating, and (**d**) located between the waveguide layer and the substrate.

In this study, dual-layer GMR structure, which is the simplest type of multi-layer GMR, was adopted, as shown in Fig. 2. The theoretical calculation was performed by dividing into four different configurations depending on the location of graphene layers. The four configurations are as follows. Configuration A: a graphene layer is located between the grating and the waveguide layers; B: a graphene layer is covering the grooves and ridges of the diffraction grating; C: a graphene layer is supported on the top surfaces of grating; D: a graphene layer is located between the waveguide layer and the substrate. The filter spectra of the proposed configurations were simulated using a two-dimensional RCWA (DiffractMOD, RSoft).

The basic structure consisted of a semi-infinite lower SiO_2_ cladding layer, a Si_3_N_4_ waveguide layer, and an upper periodic linear grating. In the initial calculation, the upper grating layer was assumed to be a monolithic grating composed of Si_3_N_4_. The number of the graphene layers was assumed to be four layers and their dielectric constant was set using Eq. (1) for in-plane direction and *ε*_z_ = 1 for out-of-plane. The unit cell size of computational simulation was *x* = −0.5 p ~ 0.5 p, *z* = −1 ~ 3 μm, and 10 harmonics were used for the calculation. It is assumed that the TE polarized light is normally incident on the filter surface. The duty cycle (=*w/p*) and thickness of the grating layer were determined by considering it as an effective medium satisfying the antireflection condition.

According to the effective medium theory (EMT), the effective refractive index of a linear diffraction grating for the TE polarized light at normal incidence was calculated using the relation given as^21^:

3 $${n}_{eff}=\sqrt{d{n}_{H}^{2}+(1-d){n}_{L}^{2}}$$

where *n*_H_ is the refractive index of the diffraction grating, *n*_L_ is the refractive index of the surrounding material, and *d* is the duty cycle. For the reference wavelength of 1.5 μm, the optimum condition for antireflection was calculated based on the Fresnel equations and found to be met when the refractive index and thickness of the effective medium were 1.374 and 0.250 μm, respectively. From this, the duty cycle of the Si_3_N_4_ grating was determined to be 0.3.

### Tunability comparison of GMR filters depending on graphene position

**Figure 3:**
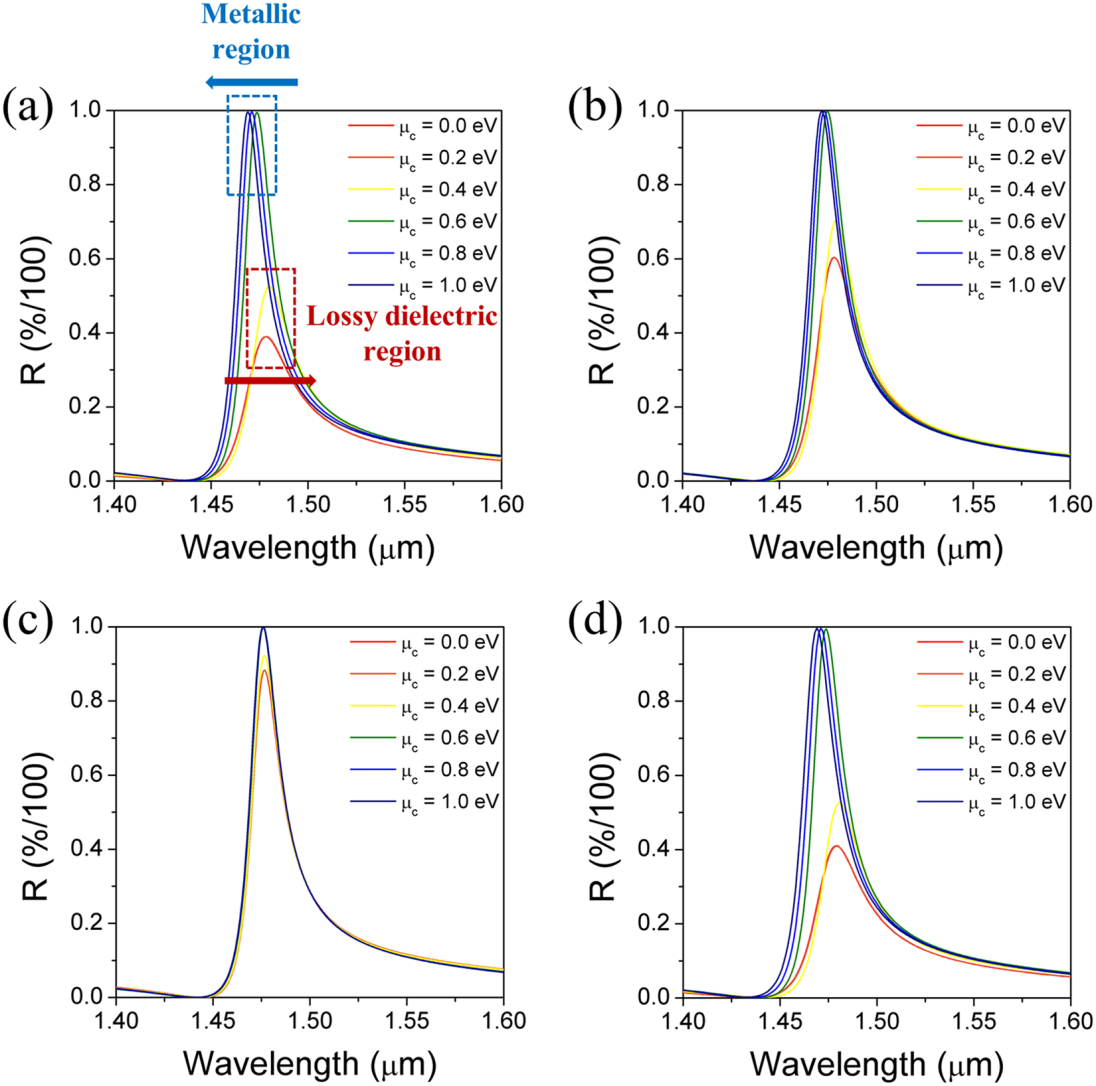
Reflection band spectra of the GMR filters as a function of chemical potential for four different configurations (**A** to **D** with Si_3_N_4_ grating). The number of graphene layers N = 4 and Γ = 0.66 meV.

Figure 3 shows the reflection band spectra of four different configurations of graphene-GMR filter calculated with varying chemical potentials of graphene. It can be seen that the shift of the resonance wavelength and the peak intensity of the reflectance depend on the location of the graphene layer, but the overall dependence on the chemical potential seems to be similar for the four different configurations. Referring to the configuration A, for the lossy-dielectric region of graphene (0 ≤ *μ*_c_ < 0.5 eV), the resonance spectrum is damped due to the optical absorption loss caused by high *ε*_2_, resulting in lower and broader reflection peak. In addition, as *μ*_c_ increases to 0.4 eV, the resonant wavelength is shifted towards the longer wavelength, which is believed to be ascribed to the increase in *ε*_1_ (Fig. 1(a)). On the other hand, in the low-loss metallic region (*μ*_c_ > 0.5 eV), the reflection spectrum of the GMR filter doesn’t seem to suffer from optical damping and the resonance wavelength was continuously blue-shifted with increasing *μ*_c_. This is due to the fact that in this region, *ε*_2_ has a negligible value close to 0, while *ε*_1_ decreases continuously with increasing *μ*_c_ of graphene.

**Figure 4:**
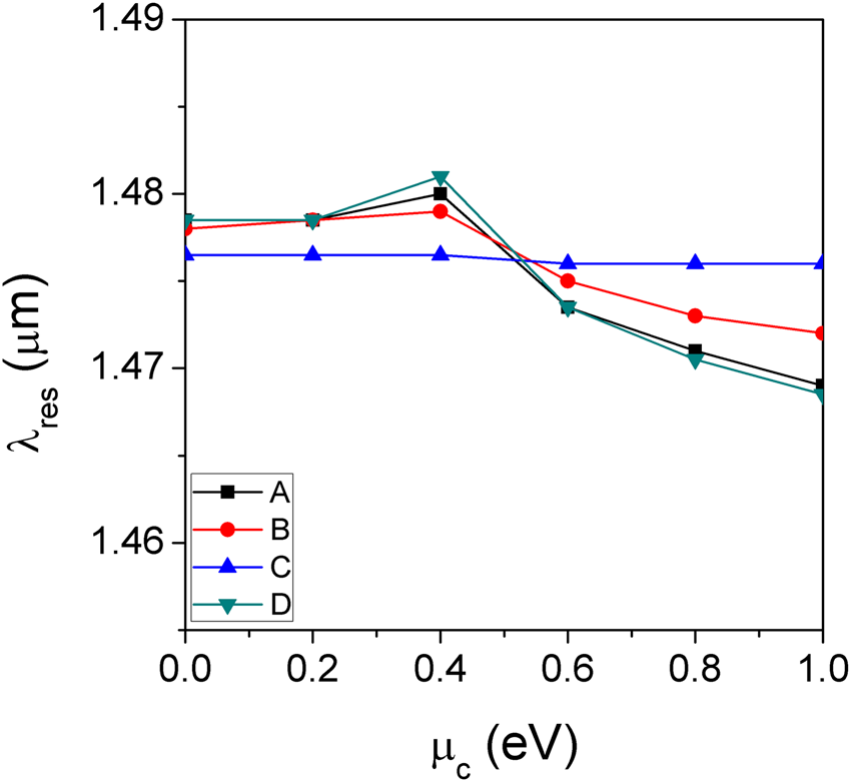
Comparison of resonance wavelength changes as a function of chemical potential between four different configurations.

The changes in resonance wavelength as a function of the chemical potential of graphene for four different configurations are compared and summarized in Fig. 4. It is found that the configuration A and D, where the graphene is in a direct contact with the Si_3_N_4_ waveguide layer, show much better tunability with the chemical potential. On the other hand, the tunability becomes smaller when the graphene is located away from the optical waveguide.

**Figure 5:**
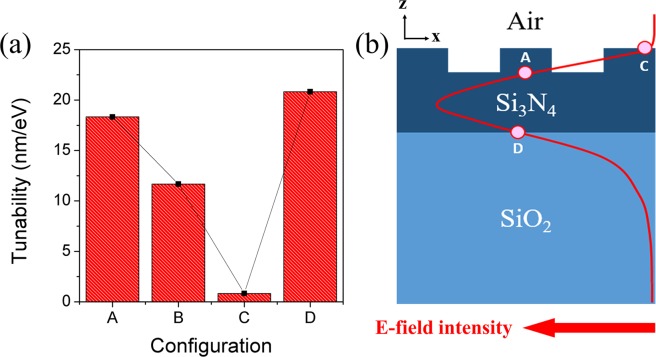
(**a**) Tunability comparison of the graphene-GMR filters with four different configurations (A to D with monolithic Si_3_N_4_ gratings) and (**b**) a schematic cross-sectional E-field intensity distribution of the monolithic grating-based GMR filter.

When the tunability of graphene-GMR filters is defined as the extent of resonance wavelength shift with respect to the unit change in chemical potential of graphene, the tunability of each configuration in the metallic region can be quantitatively compared, as shown in Fig. 5(a). The results show that the tunability obey the following relation: D ≥ A > B ≫ C. This can be understood related with the distribution of the electric-field (E-field) intensity of the optical guided-mode at each interface at which the graphene is located. Figure 5(b) depicts a schematic diagram of the spatial E-field intensity distribution in a cross-section of the monolithic grating-based GMR filter, which is the basic structure used in the calculations. Although the main portion of E-field is confined in the waveguide core, there is a certain level of leaky-field (called evanescent field) extending from the interface between the waveguide and adjacent claddings. The evanescent-field intensity at each interface increases with decreasing refractive index difference between the cladding and the waveguide, *i.e*. by making the waveguide leakier. In our model structure, the refractive index of the lower SiO_2_ cladding (${n}_{{\rm{S}}{\rm{i}}{{\rm{O}}}_{2}}=1.44$) is larger than those of the grating-air cladding (*n*_eff_ = 1.374) and the surrounding air. Thus, the refractive index difference is the smallest at the interface between the waveguide and the lower SiO_2_ cladding and the evanescent field is the largest at point D in Fig. 5(b). Consequently, the more evanescent field feels graphene and undergoes perturbation by the change in optical properties of graphene located at this point, thereby enhancing the tunability. This is largely responsible for the tunability behavior observed in Fig. 5(a).

However, as shown in Fig. 3, regardless of their configuration, since the full-width at a half-maximum (FWHM) of the reflection bands is quite broad, the tuned spectra are seriously overlapped. It should be noted that the extinction ratio, which is defined as a relative output power ratio of optical signals in a certain signal channel initially set, is inevitably small. Therefore, from the viewpoint of the extinction ratio, the overlap of the filter spectra must be reduced. The overlap of the spectra can be readily minimized by reducing the FWHM of the reflection band. One approach to narrow the bandwidth is to increase the waveguide thickness so that more E-field is confined in the waveguide core. The other is to lower the index contrast of the grating by employing a low refractive index material constituting the grating.

### Methods for improving the extinction ratio of signal channels

**Figure 6:**
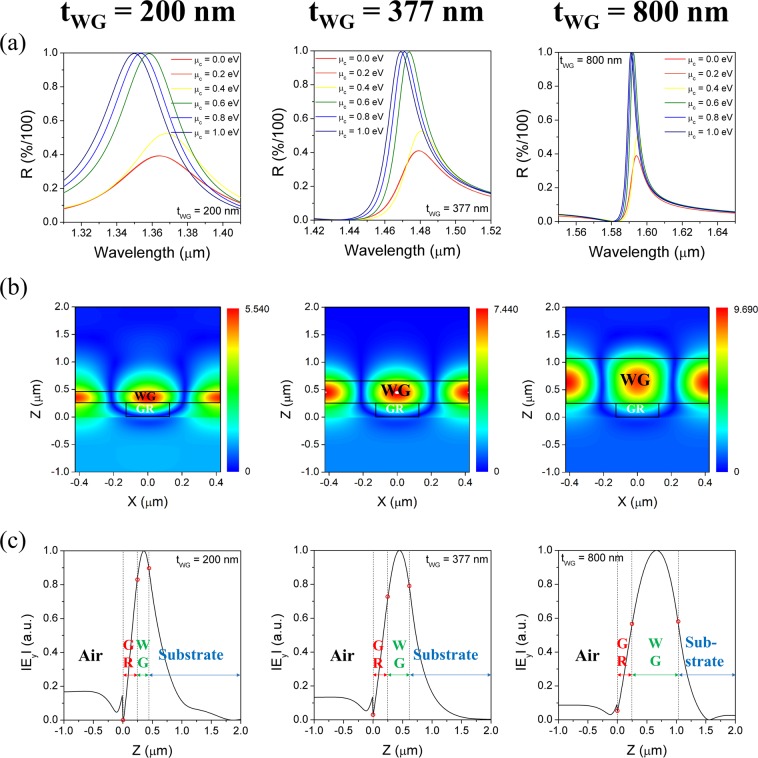
(**a**) Effect of waveguide thickness on the reflection spectra and their tuning as a function of chemical potential (for configuration D with Si_3_N_4_ grating). The number of graphene layers N = 4 and *Γ* = 0.66 meV. (**b**) Cross-sectional view of electric field distribution in x-z plane of one grating period and (**c**) E-field intensity distribution along z directions extracted from (**b**) at x = 0 for each waveguide thickness.

First, the effect of waveguide thickness on the performance of tunable GMR filter was analyzed. In the D configuration, only the thicknesses of the waveguides were varied, namely, 200, 377, and 800 nm, while the other factors remain unchanged. The calculated reflection band spectra are shown in Fig. 6(a). As the waveguide thickness increases, the resonance band occurs in the longer wavelength and the FWHM of reflection bands is significantly reduced. On the other hand, it should be noted that the tuning of the resonance band with respect to the chemical potential of graphene is reduced and the overlap issue between the tuned spectra is hardly improved. This behavior might be ascribed to the fact that with increasing waveguide thickness, the E-field confined in the waveguide core increases and hence the evanescent filed strength at the interface, at which the graphene is placed, becomes weaker. To verify this, the E-field intensity distribution in the x-z plane of the GMR filters was calculated at each resonance wavelength (Fig. 6(b)). Here, the simulation space consisted of the incident medium in the -*z* axis direction, the grating located from *z* = 0 to 0.25 μm, and the waveguide and cladding layers sequentially placed thereon. It is observed that the E-field intensity is confined in the waveguide core representing the guided-mode and the position of maximum intensity is repeated every 0.5*p*. In addition, it is noted that the maximum intensity and the portion of E-field confined in the waveguide core increases with increasing its thickness. For more quantitative comparison, the distribution of E-field intensity normalized in the z-axis direction at the center (*x* = 0) of the GMR filter was extracted from Fig. 6(b) and displayed in Fig. 6(c). The positions that the E-field intensity distribution meet the two adjacent interfaces around the waveguide is indicated by red-open dot. For the thin waveguide, the percentage of the E-field confined in the waveguide core is small, while the evanescent field at the interface becomes very strong. On the other hand, as the waveguide thickness increases, the percentage of the E-field confined in the waveguide core region significantly increases and the leaky-field intensity decreases, which is thought to lead to a deterioration in the tunability. Thus, it is confirmed that although an increase in the waveguide layer thickness is an effective way for lowering the FWHM, it adversely affects the tunability.

**Figure 7:**
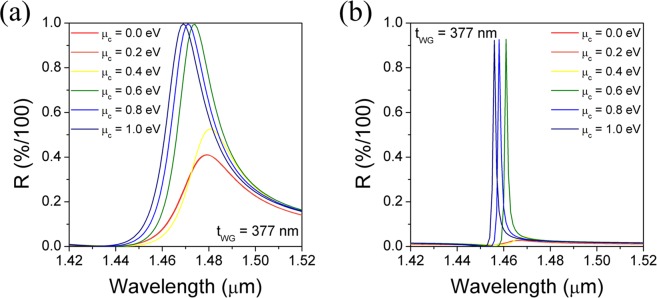
The comparison of reflection spectra of the graphene-GMR filters with (**a**) Si_3_N_4_ grating and (**b**) low-index contrast SiO_2_ grating (based on Configuration D). The number of graphene layers N = 4 and *Γ* = 0.66 meV.

Another approach to reduce the resonance linewidth is lowering the refractive index contrast of the grating by employing a grating material with low refractive index such as SiO_2_ instead of Si_3_N_4_. Since the refractive index of SiO_2_, *n*_SiO2_ = 1.47 at a wavelength of 1.5 μm, the duty cycle was estimated to be 0.765 by Eq. (3) for obtaining the same effective refractive index (*n*_eff_ = 1.374) as in the case of Si_3_N_4_ grating. The reflection band spectra of the GMR filter were calculated respectively for Si_3_N_4_ and SiO_2_ gratings in the configuration D, where all parameters were the same except for the duty cycle, as shown in Fig. 7. As compared with the case of Si_3_N_4_ grating with the same waveguide material (Si_3_N_4_) (Fig. 7(a)), use of the low-index contrast grating resulted in a dramatic narrowing in the reflection bandwidth. (Fig. 7(b)). This can be attributed to reduction in scattering of the higher order diffraction light at the grating ridge and cover medium as a result of the small difference in the refractive indices of SiO_2_ and the air^22,23^. More remarkably, the tunability of the resonance wavelength remains almost unchanged. Consequently, the overlap between the tuned spectra is significantly diminished and hence a high extinction ratio is achieved.

**Figure 8:**
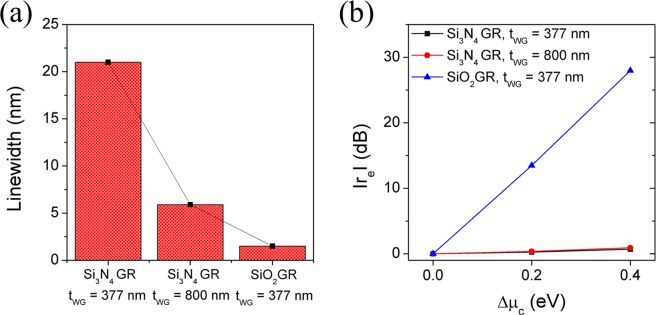
A quantitative comparison of two approaches attempted to lower bandwidth, one is to reduce the waveguide thickness and the other to employ a low-index contrast grating, in terms of (**a**) linewidth and (**b**) signal extinction ratio.

Figure 8 exhibits a quantitative comparison of the results of two approaches attempted to reduce resonance linewidth in terms of linewidth and signal extinction ratio. The basic GMR with the waveguide thickness of 377 nm and the Si_3_N_4_ grating gives a FWHM of the reflection band of about 22 nm. Their FHWMs are remarkably reduced to 6.5 and 1.5 nm, respectively (Fig. 8(a)) when the waveguide thickness increases to 800 nm or the low index contrast grating (SiO_2_) is used. On the other hand, the extinction ratio, representing a degree of output signal change with reference to a specific resonance channel (at *μ*_c_ = 0.6 eV), shows a quite different characteristic (Fig. 8(b)). Here, the extinction ratio is defined as ${r}_{e}=(-10)\log \,{R/R}_{{\mu }_{c}=0.6eV}$ and indicates the relative ratio of reflectance evaluated with varying chemical potential of graphene at the wavelength of resonance channel when *μ*_c_ = 0.6 eV. When the chemical potential increases from 0.6 to 1.0 eV, the way of increasing the waveguide thickness in the GMR filter with a high-index contrast grating such as Si_3_N_4_ suffers from the lower tunability caused by reduced leaky-field at the waveguide/cladding interface where the graphene is located, consequently resulting in the absence of improvement in the extinction ratio. However, for the GMR filter with the low-index contrast grating, the extinction ratio increases up to 27.99 dB when the baseline corrected. Therefore, these results demonstrate that use of a low-index contrast grating is an essential design rule for achieving narrower FWHM and improving the extinction ratio.

### Practical device design for experimental implementation

**Figure 9:**
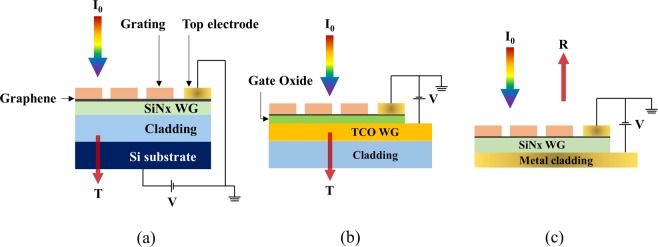
Potential back-gated tunable graphene-GMR filter configurations using (**a**) a Si substrate, (**b**) a TCO waveguide, and (**c**) a metal cladding layer as back-gate electrodes, respectively.

So far, we have explored the optimal design of optical geometry that improve the tunability and signal extinction ratio of graphene-GMR filters. On the other hand, a method of charge doping to graphene has to be provided for the practical device implementation. Graphene doping can be accomplished by optical, chemical, or electrical means. Among them, charge doping via gate voltage control has been regarded as the most convenient and effective method. Our design based on the multilayer GMR can be easily incorporated into a typical back-gated graphene field effect transistor device layout as shown in Fig. 9(a). Here, the configuration A, which is easy to fabricate, is employed and a double-side polished highly doped Si substrate, transparent in near infrared region, is used as a back gate electrode. However, in this case, the distance between the back-gate and the top electrodes is inevitably a few micrometers apart for sufficient confinement of guided mode. This causes the problem that the gate voltage to be applied needs to be excessively increased beyond several hundred volts, since the surface charge density induced in graphene, which shifts the position of the Fermi energy i.e. the chemical potential, is proportional to the electric-field strength applied between the two electrodes^24^.

As a solution to this problem, we propose two new practical device configurations for experimental implementation which drastically reduce the level of gate voltage while following the design rules developed in this work, as shown in Fig. 9(b,c) for transmissive and reflective types, respectively. The transmissive device shown in Fig. 9(b) has a configuration in which a transparent conductive oxide (TCO) is used as an optical waveguide and an additional thin gate oxide is inserted between the graphene and TCO waveguide. Since the TCO also acts as a back-gate electrode, the capacitive gap is determined only by the thickness of thin gate oxide, which contributes to substantially lowering the gate voltage to be applied as compared to the case of using a dielectric waveguide. Whereas, in a reflective type device shown in Fig. 9(c), the dielectric cladding is replaced with a metal cladding. As a result, the distance between two electrodes is reduced by the thickness of dielectric cladding removed.

To validate the proposed device configurations, RCWA simulations were performed for each model structure. For the calculation of the transmissive tunable filter with TCO waveguide, it is assumed that the cladding layer of SiO_2_ is semi-infinite and a 30 nm thick Al_2_O_3_ layer is inserted as the gate oxide. The optical constants of TCO were obtained from the Drude model^25^ calculation by setting several typical parameters, which include the electron effective mass, m^*^ = 0.3m_e_, the relaxation time, τ = 1.7 × 10^−14^ s, and the background dielectric constant, ε_∞_ = 3.6. The free electron density, n_e_ was adjusted to 1 × 10^19^ cm^−3^ to ensure good optical transmittance in the 1.5 μm wavelength region and a conductivity suitable for use as a back-gate electrode, simultaneously. The calculated refractive index of TCO was about 1.86 at 1.5 μm and its thickness was set to 250 nm. For the calculation of reflective type device, it is assumed that Si_3_N_4_ is used as the waveguide and the Au electrode is used as a metal cladding. In both transmissive and reflective type configurations, diffraction gratings made of SiO_2_ were used with their period adjusted to 950 and 960 nm, respectively. The duty cycles of the gratings were determined to be 80 and 65%, respectively, considering optimum anti-reflection effect.

**Figure 10:**
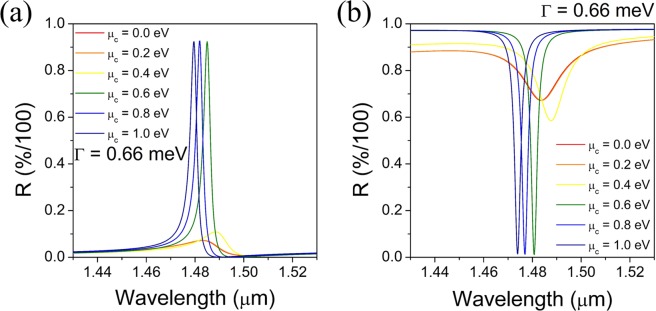
Reflection band spectra calculated as a function of chemical potential of graphene for the back-gated tunable graphene-GMR filter configurations with (**a**) a TCO waveguide and (**b**) a metal Au cladding.

The reflection band spectra of the newly proposed back-gated tunable graphene-GMR filter configurations were calculated with varying chemical potentials of graphene and shown in Fig. 10. It is obvious that both device configurations clearly share the common resonance characteristics expected by the design rules developed in this work for the multilayered GMR filters, such as a narrow bandwidth, an excellent out-of-band rejection, and a diminished level of side band, except that the reflective type device forms a reflectance dip curve. They also exhibit a band tunability very similar to and even better than the case of previous multilayer GMR configuration shown in Fig. 7(b). The slightly wider bandwidth observed may be ascribed in part to the presence of weak imaginary part of dielectric constants of TCO and Au. In the device configuration using TCO waveguide, the linewidth can be further reduced by optimizing the carrier density of TCO down to a certain level. These results demonstrate that the proposed device configurations for electric-field doping of graphene at low bias voltages provide a very promising platform for practical implementation of tunable graphene-GMR filters.

## Conclusions

In this study, a tunable GMR filter based on a large change in refractive index of graphene caused by the external stimuli was proposed. The effects of the geometrical configuration of the GMR filter and the optical properties of constituent materials including graphene on the spectral response of the filter were analyzed systematically in terms of the extinction ratio of the signal channel and the tunability. It was found that the filter displayed better tunability when the graphene was located adjacent to the waveguide and the evanescent field strength at the interface increased. However, the increase of the leaky-field, observed in the GMR filter with a thin waveguide, accompanies a line-broadening and causes the overlap between the tuned spectra to increase. Consequently, it has an adverse effect on the extinction ratio of signal. In order to reduce the linewidth of reflectance band, two approaches were proposed, i.e. thickening waveguide and employing a low-index contrast grating such as SiO_2_. The first method increased the ratio of the E-field confined in the waveguide core, thereby reducing the spectrum linewidth. However, this caused the deterioration in the tunability due to the decreased intensity of the evanescent field to be perturbed by an index modulation of graphene. Second, use of the SiO_2_ grating resulted in a high extinction ratio as a result of the narrowed linewidth of the filter spectrum without loss of tunability. Furthermore, we suggested new practical device designs that is easy to fabricate and effectively implement the graphene doping with low gate voltage, which is achievable by applying the TCO waveguide with a thin gate oxide inserted between the graphene and TCO, or by replacing the dielectric cladding with a metal one. Theoretical calculations confirm that these approaches are very effective in providing excellent tunability while significantly lowering the level of gate voltage for electric-field doping of graphene. Consequently, our results provide practical guidelines to design and implement the tunable GMR filters based on the refractive index modulation of the graphene with large and fast tunability.
